# Genetic and virulence characterisation of *Vibrio parahaemolyticus* isolated from Indian coast

**DOI:** 10.1186/s12866-020-01746-2

**Published:** 2020-03-23

**Authors:** Divya Meparambu Prabhakaran, Thandavarayan Ramamurthy, Sabu Thomas

**Affiliations:** 1 Cholera and Biofilm Research Lab, Department of Pathogen Biology, Rajiv Gandhi Centre for Biotechnology, Thiruvananthapuram, Kerala 695 014 India; 2 Centre for Human Microbial Ecology, Translational Health Science and Technology Institute, Faridabad, India

**Keywords:** *V. parahaemolyticus*, Seafood, Pandemic traits, Type 3 secretion system, Pulsed-field gel electrophoresis, Cytotoxicity, Invasion

## Abstract

**Background:**

*V. parahaemolyticus* is autochthonous to the marine environment and causes seafood-borne gastroenteritis in humans. Generally, *V. parahaemolyticus* recovered from the environment and/or seafood is thought to be non-pathogenic and the relationship between environmental isolates and acute diarrhoeal disease is poorly understood. In this study, we explored the virulence potential of environmental *V. parahaemolyticus* isolated from water, plankton and assorted seafood samples collected from the Indian coast.

**Results:**

Twenty-two *V. parahaemolyticus* isolates from seafood harboured virulence associated genes encoding the thermostable-direct haemolysin (TDH), TDH-related haemolysin (TRH), and Type 3 secretion systems (T3SS) and 95.5% of the toxigenic isolates had pandemic strain attributes (t*oxRS*/new^+^). Nine serovars, with pandemic strain traits were newly identified and an O4:K36 *tdh*^−^*trh*^+^*V. parahaemolyticus* bearing pandemic marker gene was recognised for the first time. Results obtained by reverse transcription PCR showed *trh*, T3SS1 and T3SS2β to be functional in the seafood isolates. Moreover, the environmental strains were cytotoxic and could invade Caco-2 cells upon infection as well as induce changes to the tight junction protein, ZO-1 and the actin cytoskeleton.

**Conclusion:**

Our study provides evidence that environmental isolates of *V. parahaemolyticus* are potentially invasive and capable of eliciting pathogenic characteristics typical of clinical strains and present a potential health risk. We also demonstrate that virulence of this pathogen is highly complex and hence draws attention for the need to investigate more reliable virulence markers in order to distinguish the environmental and clinical isolates, which will be crucial for the pathogenomics and control of this pathogen.

## Background

*Vibrio parahaemolyticus* is a Gram-negative, halophilic bacterium that inhabits marine and estuarine environments. Since its discovery in 1950 as the causative agent of gastroenteritis in Japan [1], *V. parahaemolyticus* has become one of the leading causes of food-borne illness in humans. Infection is closely associated with the consumption of raw or undercooked seafood, resulting in self-limiting diarrhoea [2]. Rarely, *V. parahaemolyticus* also causes wound infections and septicaemia [2]. The virulence of *V. parahaemolyticus* is associated with the production of thermostable-direct haemolysin (TDH) (encoded by *tdh* gene) and/or TDH-related haemolysin (TRH, encoded by *trh* gene) as well as two type III secretion systems (T3SS) [3–5]. Two sets of the genes T3SS1 and T3SS2 are present on chromosomes 1 and 2, respectively [5].

Though a majority of clinical *V. parahaemolyticus* generally carry *tdh* and/or *trh*, only a small proportion of environmental isolates have been found to harbour the hemolysin genes [6–8]. T3SS1 that produce cytotoxicity is present in all *V. parahaemolyticus* isolates irrespective of their source. T3SS2, on the other hand, is both cytotoxic and enterotoxic and has two phylogroups; T3SS2α that co-localise with *tdh* and T3SS2β found in association with *trh* [9]. The environmental population of *V. parahaemolyticus* is increasingly acquiring the virulence-related genes that classically define a clinical isolate [10, 11]. However, the true pathogenic potential of such strains has not been evaluated at large.

After the emergence of the *V. parahaemolyticus* serovar O3:K6 in 1996 from Kolkata, India [12], infections caused by several serovariants, collectively called pandemic strains, have increased due to their global spread with several diarrhoeal epidemics [13]. The pandemic clone has the typical *tdh*^+^*trh*^−^, *toxRS*/new^+^, *orf8*^+/−^ genotype, which is identified by Group-specific PCR (GS-PCR) that detects the sequence variation in *toxRS* gene (t*oxRS*/new) [14, 15]. The burden of *V. parahaemolyticus* diarrhoea is very high in Asian countries [16–18] and the outbreaks are related to raw shellfish consumption in the United States and Canada [19, 20]. In addition, sporadic outbreaks have been reported in coastal Europe [21, 22]. Although the pandemic strains are still prevalent in diarrhoeal cases in India [23], there is a paucity of information on *V. parahaemolyticus* from different environmental sources.

The aim of this study was to examine *V. parahaemolyticus* from environmental and seafood samples collected along the southern Indian coast for serogroup, putative virulence and their pathogenic potential on intestinal epithelial cell line.

## Results

### Identification of *V. parahaemolyticus* from the environment

Four hundred and seventeen *V. parahaemolyticus* isolates were identified from environmental and seafood samples collected through five districts of the coastal belt of Kerala during the sampling period. The highest recovery of the organism was from seafood (225/417, 53.9%) followed by water (152/417, 36.5%) and plankton (40/417, 9.6%). Environmental parameters such as temperature, pH and salinity were not checked during sampling, as the objective was focused mainly towards genetic profiling and pathogenic potential of *V. parahaemolyticus.*

### Distribution of virulence genes

The isolates were screened for 22 virulence markers, including genes encoding haemolysins, T3SS1, T3SS2α, and T3SS2β and the pandemic strain marker of ORF-8 and group-specific PCR (GS-PCR). Twenty two (5.3%) isolates were found to contain either of the haemolysins and were potentially toxigenic (Table 1); 19 had *tdh* (*tdh*^+^*trh*^−^) while 3 had *trh* (*tdh*^−^*trh*^+^) and all the isolates represented seafood (22/225, 9.8%). None were found to be *tdh*^+^*trh*^+^.

**Table 1:** Characteristics of 22 environmental *V. parahaemolyticus* isolates collected from the south-west coast of India

Strain Id	Serotype	***toxR***	***tlh***	***tdh***	***trh***	***toxRS***/new	***orf8***	T3SS2 genes	
H1	O1:K17	+	+	+	–	+	–	*vopC*, *vopT*, *vscC2*, *vopA*, *vopB2*, *vopL*	T3SS2α
H2	OUT:KUT	+	+	+	–	+	–	*vopC*, *vopZ*, *vopL*	
H3	O5:KUT	+	+	+	–	+	–	*vopC*, *vopT*, *vscC2*, *vopA*, *vopB2*	
H4	O5:K17	+	+	+	–	+	–	*vopC*, *vopZ*, *vopA*, *vopL*	
H5	O1:K19	+	+	+	–	+	–	*vopC*, *vopT*	
H6	O1:K25	+	+	+	–	+	–	*vopC*, *vopT*, *vopZ*	
H7	O1:K25	+	+	+	–	–	–	*vopC*, *vopT*, *vopZ*	
H8	O1:K23	+	+	+	–	+	–	*vopC*, *vscC2*, *vopB2*, *vopL*	
H9	O1:K25	+	+	+	–	+	–	*vopC*, *vscC2*, *vopB2*, *vopL*	
H10	O5:K17	+	+	+	–	+	–	*vopC*, *vopZ*, *vscC2*, *vopA*, *vopB2*	
H11	O1:K25	+	+	+	–	+	–	*vopC*, *vopZ*, *vscC2*	
H12	O10:K24	+	+	+	–	+	–	*vopC*, *vopZ*, *vopB2*	
H13	O2:KUT	+	+	+	–	+	–	*vopC*, *vopZ*, *vopB2*, *vopL*	
H14	O4:K29	+	+	+	–	+	–	*vopC*, *vopZ*, *vscC2*, *vopB2*, *vopL*	
H15	O5:K17	+	+	+	–	+	–	*vopC, vopB2*	
H16	O1:KUT	+	+	+	–	+	–	*vopC*, *vopB2*, *vopL*	
H17	O5:K20	+	+	+	–	+	–	*vopC*, *vopT*, *vopB2*	
H18	O5:K20	+	+	+	–	+	–	*vopC*, *vopA*, *vopB2*	
H19	O4:K42	+	+	+	–	+	–	*vopC*, *vopA*, *vopB2*, *vopL*	
C12	O3:KUT	+	+	–	+	+	–	*vscC2*, *vopB2*, *vscS2*, *vopC*, *vopA*, *vopL*	T3SS2β
C13	O3:KUT	+	+	–	+	+	–	*vscC2*, *vopB2*, *vscS2*, *vopC*, *vopA*, *vopL*	
K23	O4:K36	+	+	–	+	+	–	*vscC2*, *vopB2*, *vscS2*, *vopC*, *vopA*, *vopL*	

The toxigenic isolates were then checked for T3SS genes coding for effectors and apparatus proteins. T3SS1 (*vscP*, *vopS*, *vscK*, *vscF* and *VPA0450*) was present in all the 22 isolates. Several genetic elements of T3SS2α were detected with isolates harbouring more than three genes. While *vopC*, thought to mediate invasion of non-phagocytic cells, was present in 19 *tdh*^+^*trh*^−^ isolates, *vopB2* was detected in 13, *vscC2* in 7, *vopT*, *vopL* and *vopA*/*P* in 5, 9 and 6 isolates, respectively. Six of the isolates also harboured *vopZ*, the gene responsible for intestinal colonisation and enterotoxocity. Genes encoding the T3SS2β (*vscC2*, *vopB2*, *vscS2*, *vopC*, *vopA*/*P* and *vopL*) were present in all the three *tdh*^−^*trh*^+^ isolates (Table 1). Twenty one isolates (18 *tdh*^+^*trh*^−^, 3 *tdh*^−^*trh*^+^) belonged to the pandemic strain type (*toxRS*/new^+^).

### PFGE analysis of toxigenic strains

The 22 toxigenic isolates consisted of 15 serovars with combinations of six O groups (O1, O2, O3, O4, O5 and O10) and nine different K types (K17, K19, K20, K23, K24, K25, K29, K36 and K42). The predominant serovar was O1:K25 (*n* = 4). Two *tdh*^−^*trh*^+^ isolates belonged to O3:KUT while the other was O4:K36.

**Fig. 1:**
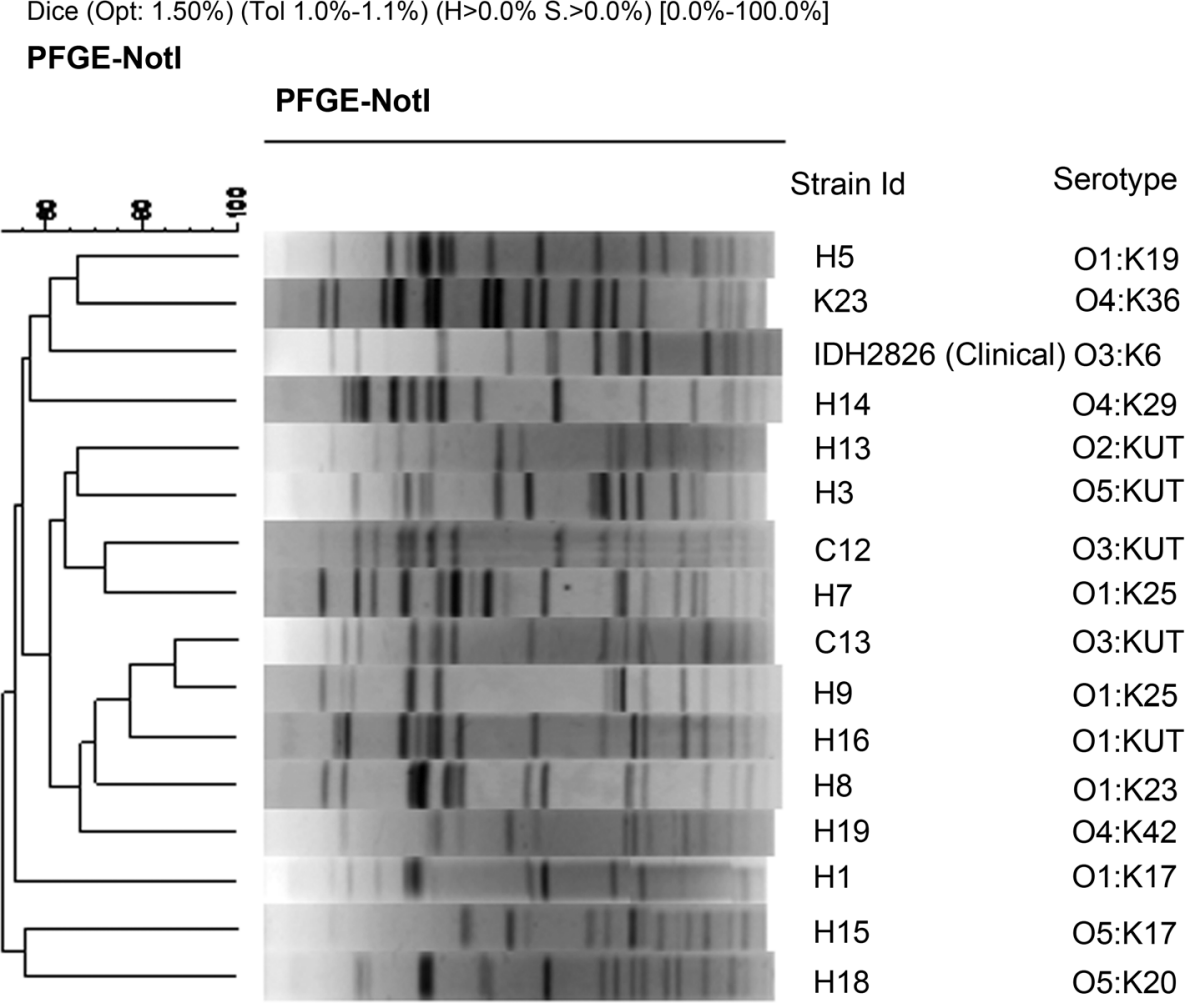
*No*tI digested PFGE profile of *V. parahaemolyticus* with dendrogram. Clustering was performed using the unweighted pair group method (UPGMA) and the Dice correlation coefficient with a position tolerance of 1.0%

Based on the serovar profile, a total of 15 isolates were analysed by PFGE and the pattern compared to an O3:K6 clinical isolate (NICED, Kolkata, India). The minimum genetic similarity of the isolates was 55% and fell into four distinct clusters (Fig. 1). Those that clustered together at 60% similarity with the O3:K6 were isolates of O1:K19, O4:K29 (*tdh*^+^*trh*^−^) and O4:K36 (*tdh*^−^*trh*^+^) serovars. It was observed that isolates of the same O1:K25 serovar clustered differently. Likewise, the three *tdh*^−^*trh*^+^ isolates possessing identical virulence profiles were present in three different clusters. Interestingly, the GS-PCR positive and negative isolates clustered together. Overall, the results revealed a high genetic variability in environmental isolates of *V. parahaemolyticus*.

### Selection of strains for pathogenicity studies

A total of 12 isolates of *V. parahaemolyticus* including 8 environmental and 4 clinical were included in downstream assays (Table 2). The environmental strains were selected based on the T3SS profile and serovar type.

**Table 2:** *V. parahaemolyticus* strains used for pathogenicity studies

Group	Strain Id	Serogroup	Toxin Profile	Source
Environmental, toxigenic	H10	O5:K17	*tdh*^+^*trh*^−^	Present study, seafood
	H12	O10:K24	*tdh*^+^*trh*^−^	
	H14	O4:K29	*tdh*^+^*trh*^−^	
	K23	O4:K36	*tdh*^*−*^*trh*^*+*^	
Environmental, non-toxigenic	AJM1	O5:K17	*tdh*^*−*^*trh*^−^	Present study, water
	ME1	O10:KUT	*tdh*^*−*^*trh*^−^	
	ME4	O1:K32	*tdh*^*−*^*trh*^−^	
	ME8	O1:K32	*tdh*^*−*^*trh*^−^	
Clinical	RIMD2210633	O3:K6	*tdh*^+^*trh*^−^	Reference pandemic isolate, Japan
	AP11243	O1:KUT	*tdh*^+^*trh*^−^	Bangladesh
	IDH03525	O3:K6	*tdh*^+^*trh*^−^	India
	ATCC17802	O1:K1	*tdh*^*−*^*trh*^*+*^	Japan

### Expression of virulence genes

Expression of virulence genes *tdh*, *trh*, effectors of T3SS1 (*vopS*, *VPA0450*), T3SS2α (*vopC*, *vopT*, *vopZ*, *vopA*/*P*, *vopL*) and T3SS2β (*vopC*, *vopA*/*P*, *vopL*) was determined by RT-PCR in relation to *gyrB*, which is a constitutively expressed housekeeping gene. The *tdh* gene was transcribed by all the *tdh*^+^*trh*^−^ clinical isolates, whereas inconsistent results were observed with the seafood isolates H10, H12 and H14. Either the bands were very faint or not detected at all in repeated PCRs. It was concluded that the *tdh* gene was not functional in the seafood isolates. In contrast, the *tdh*^−^*trh*^+^ isolate K23 and its clinical counterpart ATCC17802 transcribed *trh* gene with the production of the corresponding amplified cDNA (Additional file 1: Figure S1).

The expression results were verified by the haemolytic activity of isolates on human RBC (Additional file 1: Figure S2). Both the *tdh*- and *trh*- containing seafood strains showed extremely weak haemolytic activity (≤5%) after 6 h of incubation, whereas the clinical isolates were 50% haemolytic after 6 h, except ATCC17802 that could lyse only 3% of RBC. The haemolytic action of the seafood isolates showed only a modest increase when incubation was extended till 12 h while a dramatic rise (> 90%) was seen with *tdh*^+^*trh*^−^ clinical isolates except *tdh*^−^*trh*^+^ ATCC17802 (20%).

All the selected seafood isolates irrespective of the toxin type, transcribed T3SS1 effectors indicating they are functional (Additional file 1: Figure S3). Prominent amplification of T3SS2β genes was also detected in the isolate K23 (Additional file 1: Figure S3). On the other hand, T3SS2α effectors were not transcribed by any of the *tdh*^+^*trh*^−^ seafood isolates. Surprisingly, a faint band was detected for *vopV* in the isolate H14, which was absent in the control RNA (Additional file 1: Figure S3). As expected, the clinical control isolates functionally expressed the T3SS genes.

### Cytotoxicity of environmental *V. parahaemolyticus*

**Fig. 2:**
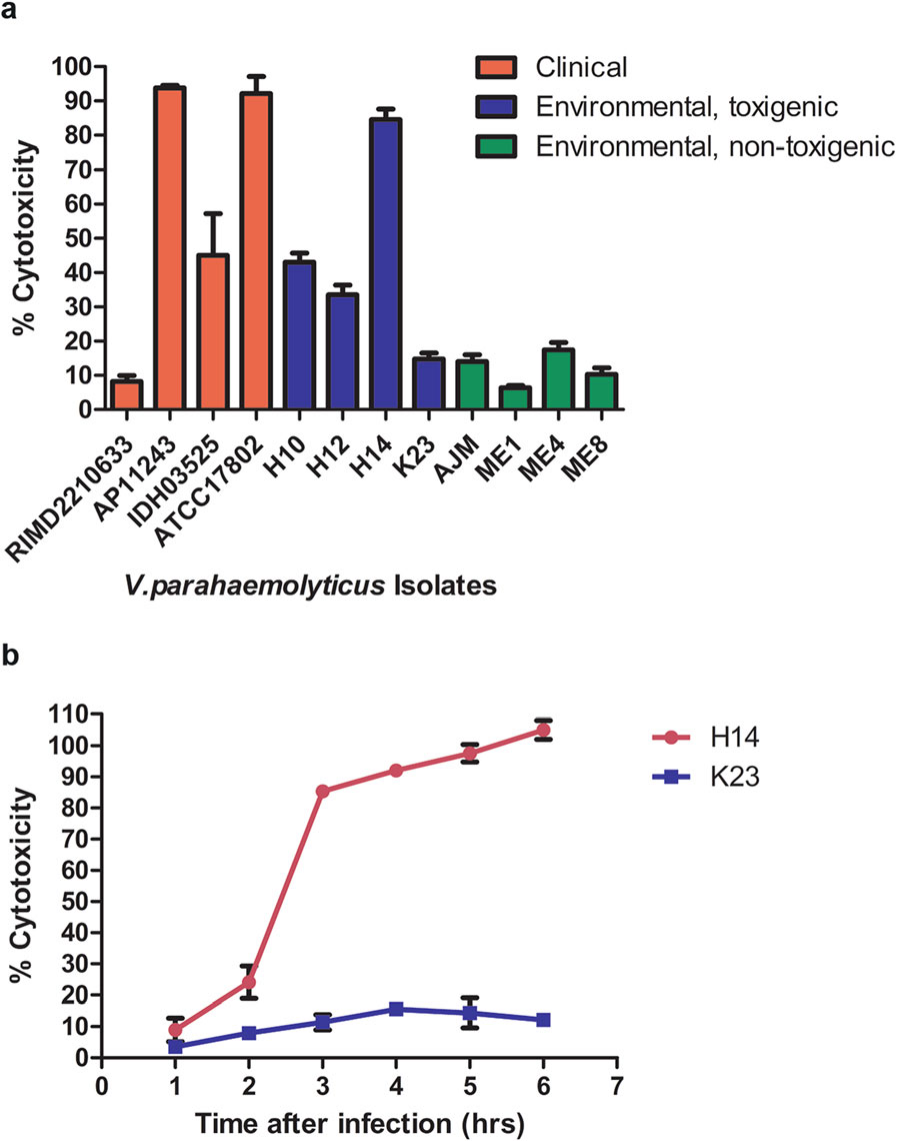
LDH cytotoxicity assay. **a** Percentage of cytotoxic activity, as measured by LDH released from Caco-2 cells infected with clinical and environmental *V.parahaemolyticus* strains. The results represent the means of three independent determinations, performed six times ± SE. **b** Time course analysis of cytotoxicity induced by H14 and K23. The results represent the means of two independent determinations ± SE, each performed in triplicate

Cytotoxicity was tested by measuring the levels of cytoplasmic LDH released into the medium during infection of Caco-2. This release indicated the degree to which the integrity of host cell membrane was compromised. Only two clinical isolates, AP11243 (*tdh*^+^*trh*^−^) and ATCC17802 (*tdh*^−^*trh*^+^) were highly cytotoxic to Caco-2 cells (94% and 93%, respectively) while the reference strain RIMD2210633 showed a mean cytotoxicity of 8% (Fig. 2a). Among *tdh*^+^*trh*^−^ seafood isolates, H10 and H12 showed good cytotoxicity (43% and 34%, respectively), whereas the isolate H14 was cytotoxic to 85% of cells, almost comparable to that of AP11243. The *tdh*^−^*trh*^+^ isolate K23 that had a fully functional T3SS exhibited low cytotoxic activity (15%). Very low cytotoxic activity, ranging from 6 to 17%, was shown by the four non-toxigenic isolates. However, when compared, the results between clinical, environmental toxigenic and non-toxigenic isolates were not statistically significant (*p* > 0.05, one-way ANOVA). A time course analysis of LDH release by *tdh*^+^*trh*^−^ H14 and *tdh*^−^*trh*^+^ K23 was done up to 6 h post-infection. There was a constant increase in cytotoxicity for H14 with 100% cell lysis at 6 h while the cytotoxicity induced by K23 reached a peak at 4 h (15%) and slightly decreased thereafter (Fig. 2b).

### Adhesion and invasion

**Fig. 3:**
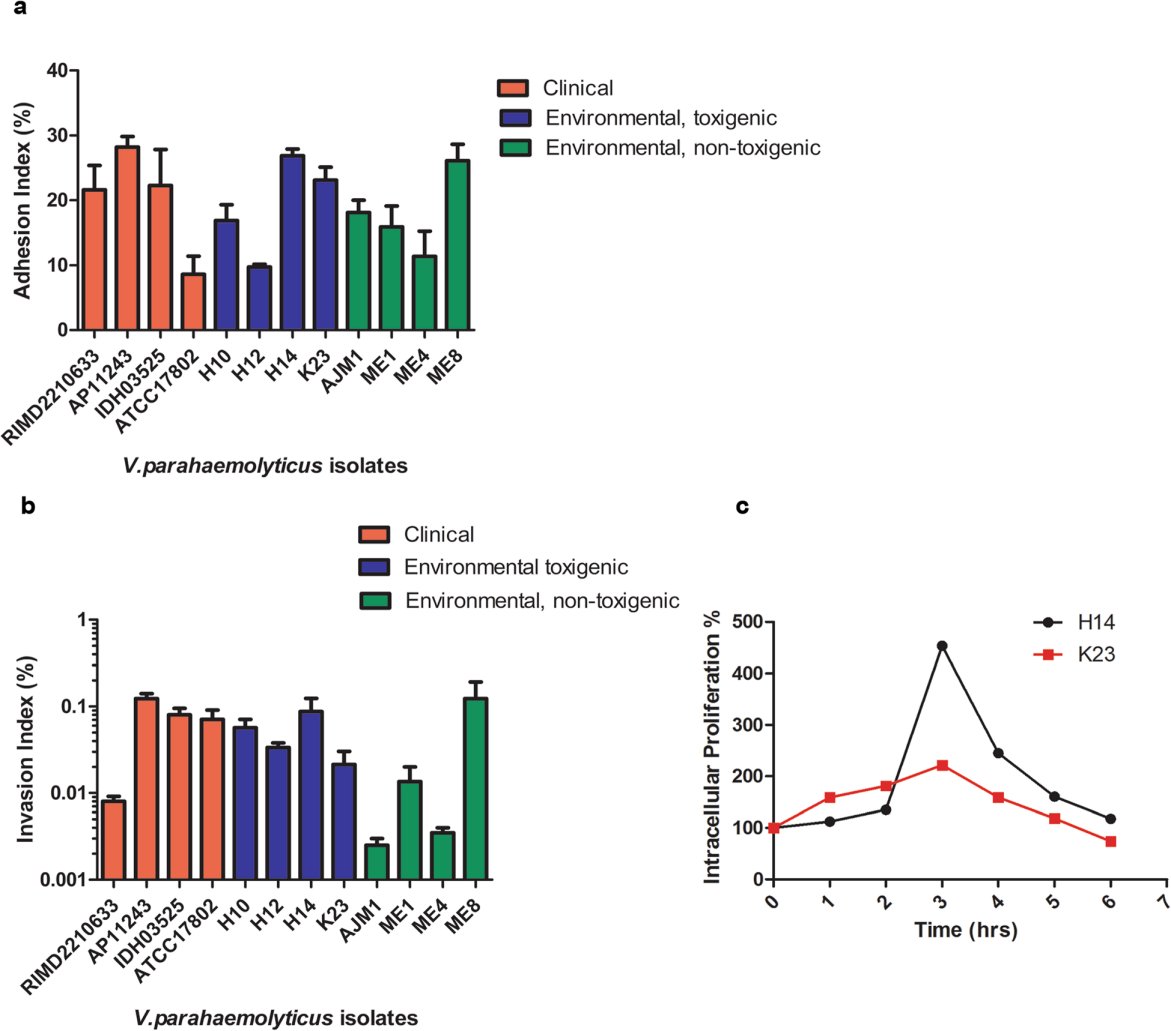
Adhesion (**a**) and invasion (**b**) index of *V. parahaemolyticus* in Caco-2 cells. The values are means ± SE of three independent experiments, each performed in triplicate. Adhesion and invasion indices between groups are not statistically significant (*p* > 0.05). **c** Intracellular proliferation assay of *V. parahaemolyticus* H14 and K23 in Caco-2 cells

*V. parahaemolyticus* contains several adhesins to promote attachment and subsequent colonization of host cells. Since it is a key event for the successful establishment of infection, ability of the environmental isolates to adhere to Caco-2 cells were determined and compared to the clinical isolates. The adhesion of the *V. parahaemolyticus* isolates to Caco-2 cells ranged from 8 to 28% (Table 3, Fig. 3a). All the *tdh*^+^*trh*^−^ clinical isolates showed good adhesion ability of ≥22%, except ATCC17802 (9%). Irrespective of the toxigenic profile, the environmental isolates exhibited more than 15% adhesion to Caco-2 cell with K23, H14 and ME8 (non-toxigenic) showing the maximum adhesion potential of 23, 27 and 26%, respectively. This shows that environmental isolates adhered firmly to intestinal epithelial cells. There was no difference in adhesion among *tdh*^+^ and *trh*^+^ pathotypes (*p* > 0.05, Student’s t-test) as well as between the different groups (*p* > 0.05, one-way ANOVA).

**Table 3:** Adhesion and invasion indices of *V. parahaemolyticus* isolates

Strain ID	Average ± SE (%)	
	Adhesion Index	Invasion Index
RIMD2210633	21.6 ± 3.79	0.008 ± 0.001
AP11243	28.2 ± 1.66	0.12 ± 0.018
IDH03525	22.3 ± 5.54	0.08 ± 0.015
ATCC17802	8.62 ± 2.81	0.071 ± 10.02
H10	16.9 ± 2.41	0.06 ± 0.015
H12	9.75 ± 0.25	0.034 ± 0.005
H14	26.9 ± 1.02	0.087 ± 0.037
K23	23.1 ± 2.03	0.021 ± 0.009
AJM	18.1 ± 1.9	0.003 ± 0.001
ME1	15.95 ± 3.15	0.014 ± 0.007
ME4	11.4 ± 3.9	0.004 ± 0.001
ME8	26.1 ± 2.55	0.12 ± 0.069
*S.* Typhimurium	30.8 ± 2.85	1.185 ± 0.665
*E. coli* JM109	6.19 ± 1.89	0.004 ± 0.0005

The ability of the selected *V. parahaemolyticus* isolates to invade Caco-2 cells was determined using the classical gentamicin protection assay. Before the experiment, gentamicin MIC of all the isolates was determined using E-test (MIC ≤4 μg/ml).

Among the clinical isolates, AP11243 displayed the highest mean invasion index (0.12%) followed by ATCC17802 (0.071%) and IDH03525 (0.08%) while RIMD2210633 showed very low invasion potential of 0.008% (Table 3, Fig. 3b). Of the toxigenic environmental isolates, 0.087% of H14 could invade Caco-2 cells followed by H10 and H12. Again, K23, with a fully functional *vopC* (mediates invasion of non-phagocytic cells) displayed a lower invasion potential (0.021%) compared to *tdh*^+^*trh*^−^ counterparts. Among the *tdh*^−^*trh*^−^ isolates, it was surprising to see the invasion index of ME8 was equal to that of AP11243 (0.12%). The rest of the isolates were not invasive, except for ME1, which showed a slight invasion, when compared with the non-invasive control *E. coli* JM109 (Table 3). Here also, the results were not statistically significant between the various groups under study (*p* > 0.05).

An intracellular proliferation assay was done subsequently to know whether the toxigenic environmental isolates could persist and replicate intracellularly following invasion into the Caco-2 cells. The experiment was done with the highly cytotoxic, adherent and invasive H14 (*tdh*^+^*trh*^−^) and K23 (*tdh*^−^*trh*^+^), which has shown a lesser pathogenic potential in spite of having a complete T3SS. H14, and to a lesser extent, K23 rapidly proliferated inside Caco-2 cells (Fig. 3c). Peak numbers of H14 (450% of initial intracellular CFU) was observed in about 3 h. This number decreased rapidly and intracellular bacteria were still visible at 6 h of infection even though the levels were similar to that detected upon initial enumeration (~ 100%). Intracellular K23 also peaked at 3 h of infection, but the number of bacteria was lower (220% of initial intracellular cfu) and at the end of 6 h, the numbers declined. The decrease in intracellular bacteria could be attributed to the lysis of eukaryotic cells and subsequent killing in the gentamicin containing medium. We did observe rounding of cells by 2 h of incubation and detachment of cells from the 24-well plates by 6 h, which made it impossible to prolong the experiment any further.

### Alteration of ZO-1 and F-actin organisation by environmental *V. parahaemolyticus*

**Fig. 4:**
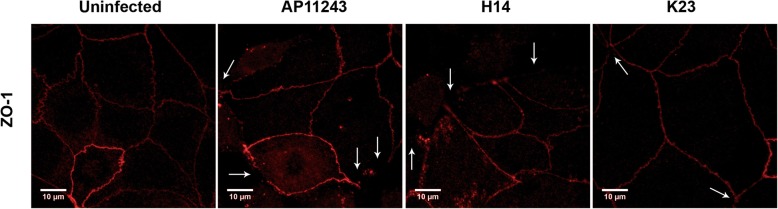
ZO-1 redistribution in Caco-2 cells caused by *V. parahaemolyticus* environmental strains after 2 h of infection. Micrographs are representative of two separate experiments

*V. parahaemolyticus* has been known to compromise epithelial barrier integrity and associated disruption of actin and the tight junction proteins [24, 25]. The effect of H14 and K23 on the localization of ZO-1 (a peripheral tight junction protein), and filamentous actin was examined using confocal microscopy. AP11243 was used as positive control strain as it showed more cytotoxicity and invasion potential than RIMD2210633. In uninfected cells, ZO-1 was located at the periphery and appeared as a continuous brightly stained band with a typical ‘honey-comb structure’ or ‘chicken-wire pattern’ revealing intact boundaries (Fig. 4). In cells infected with AP11243, there was clear disruption of the monolayer, with visible strand breaks and punctate discontinuity. Infection with *tdh*^+^*trh*^−^ H14 isolate elicited a stronger disruption of ZO-1; large areas were seen where ZO-1 was completely displaced. In contrast, the *tdh*^−^*trh*^+^ K23 infected cells showed smaller but visible breaks, although the cell membrane integrity was preserved.

**Fig. 5:**
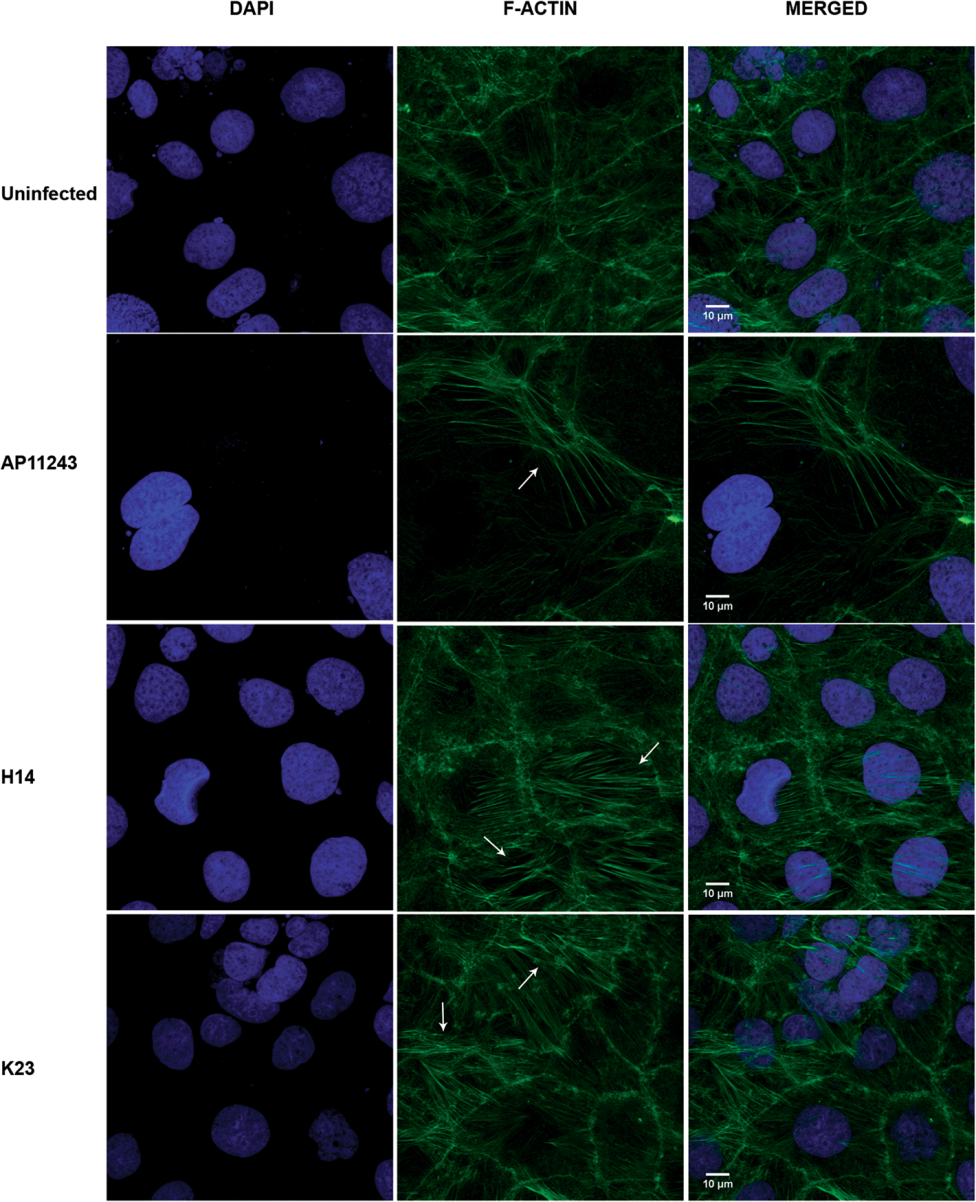
Reorganization of filamentous actin following infection with *V. parahaemolyticus*. Confluent Caco-2 cells were infected with *V. parahaemolyticus* AP11243, H14 and K23 for 2 h and double labelled with Alexa Fluor 488 Phalloidin (F-actin, green) and DAPI (nucleus, blue). Micrographs are representative of two different experiments

Actin filaments were visualised by fluorescently labelled bicyclic peptide, phalloidin that selectively binds to F-actin. Uninfected cells had branched, reticular actin network without any particular orientation. In contrast, AP11243 induced bundling of actin into long, parallel stress fibres. H14 infected cells had more number of such parallel arrays of F-actin. Surprisingly, cells infected with K23 depicted more prominent actin bundles that spanned the entire cell (Fig. 5).

## Discussion

The present study examined the distribution of virulence related genes in 417 *V. parahaemolyticus* isolated from the environment and seafood and focused on *tdh* and/or *trh* isolates to determine the pathogenicity. An abundance of *V. parahaemolyticus* was seen in the water compared to plankton. This was in contrast to other studies where higher densities of *V. parahaemolyticus* were obtained from plankton [26, 27]. However, higher numbers of the bacterium were found in the Georgian coastal waters [28]. More than 5% of the *V. parahaemolyticus* isolates were potentially toxigenic, based on the presence of *tdh* and *trh*, of which *tdh* was detected in a higher number of isolates (4.6%). Previous studies from the south-west coast of India have reported a higher detection of *trh*-positive *V. parahaemolyticus* especially after 18 h of enrichment [29, 30]. The toxigenic *V. parahaemolyticus* isolates obtained in this study were identified exclusively from seafood and none of the water and plankton isolates harboured the haemolysin genes. Hemolysin gene identification is partly dependent on the detection technique employed. The use of modified primers could recover 48 and 8% *tdh* and *trh* bearing isolates respectively from an estuarine system [31]. In another study, DePaola et al., tested a large number of colonies from oysters by DNA probes against *tlh* and *tdh* and found that 21.8% of them were pathogenic [6]. Similarly, real time PCR could detect 20 and 40% more *tdh* and *trh* positive *V. parahaemolyticus* from oyster samples and 13 and 40% from water samples compared to the conventional techniques [32]. Thus, detection of toxigenic *V. parahaemolyticus* from environmental samples appears to be influenced by several factors.

T3SSs are an important determinant of pathogenicity of *V. parahaemolyticus*. All the toxigenic *V. parahaemolyticus* strains in this study carried the T3SS1 genes, consistent with previous reports [17, 33]. The distribution of T3SS2α genes was highly diverse with the loss of at least one gene from the *tdh*^+^*trh*^−^ isolates. Some of the isolates showed weak amplification with the T3SS2α primer pair or additional primers designed. Jones et al. had also reported anomalies in the T3SS2α gene amplification [33]. This suggests high sequence variability in environmental isolates or an absence of these genes due to partial uptake of VPaI during horizontal transfer. Further, *vopB2* was present in 68.4% of *tdh*^+^*trh*^−^ isolates in contrast to earlier findings that it is associated with clinical and not environmental isolates [33, 34]. Some recent reports have documented the presence of *vopB2* in environmental isolates [35, 36]. Hence, *vopB2* may not be a good indicator of virulence as originally suggested [34]. About 33% of *tdh*^+^*trh*^−^ isolates harboured *vopZ* gene that encodes the effector, VopZ, known for *V. parahaemolyticus* induced diarrhoea and intestinal pathology. To the best of our knowledge, this is the first study to document the presence of *vopZ* (VPA1336) in *V. parahaemolyticus* isolated from seafood.

Majority of the toxigenic isolates (95.5%) had pandemic strain features (*toxRS*/new^+^). Of these, nine serovars (O1:K17, O1:K19, O1:K23, O10:K24, O2:KUT, O4:K29, O5:K20, O4:K42 and O4:K36) were newly identified with pandemic strain traits. Another striking observation is the appearance of *tdh*^−^*trh*^+^ isolates carrying the *toxRS*/new^+^ pandemic marker. Pandemic strains of *V. parahaemolyticus* are mostly associated with the *tdh* gene with few reports of GS-positive *trh*-containing isolates from clinical cases [23, 37, 38]. Data pertaining to the pandemic status of environmental *trh*-positive *V. parahaemolyticus* is lacking. Thus, this study is the first to report the isolation of a new serovar (O4:K36) of *tdh*^−^*trh*^+^*V. parahaemolyticus* from seafood with potential pandemic traits. Currently the pandemic group is defined by the presence of *tdh* and *toxRS*/new genes. So, the pandemic status and evolution of the three *tdh*^−^*trh*^+^ isolates identified in this study should be confirmed employing other genetic markers including genomic islands VPaI-1, VPaI-4 and VPaI-5 that are specific for the pandemic group [39–41] as well as whole genome sequence analysis. Besides, our finding of *tdh*^−^*trh*^+^ O3:KUT serovar and its recent isolation from acute diarrhoea in India [23] underscores better cognizance of an epidemiological association between environmental and clinical strains.

Though considered a “gold standard”, it is only recently that PFGE has been extensively employed for subtyping environmental strains of *V. parahaemolyticus* [8, 42, 43]. The PFGE pattern of the 15 isolates of this study showed considerable diversity and the new serovars having pandemic attributes were not related to the pandemic O3:K6 isolate. Further, it was noted that strains with identical serotype and/or genotype had different PFGE profiles, as in the case of *tdh*^+^*trh*^−^ isolates. Moreover, one of the O1:K25 isolate which was not positive in the GS-PCR, clustered along with the isolates having pandemic strain traits. A similar pattern was observed during molecular typing of *V. parahemolyticus* isolates in China, where seafood isolates having a varying combination of *tdh*, *trh* and *toxRS*/new genes clustered together with pandemic isolates whereas a *tdh*^+^*trh*^−^, *toxRS*/new^+^ clinical isolate was not genetically related to the pandemic clones [44]. Comparison of MLST datasets of different bacterial species revealed environmental isolates of marine and estuarine *V. parahaemolyticus* and *V. vulnificus* have very high homologous recombination rate as they frequently adapt for survival [45]. It was proposed that *V. parahaemolyticus* may have an epidemic population structure [46] where the isolates undergo frequent genetic recombination resulting in well-adapted clones [47]. Considering the increase in environmental strains acquiring virulence and pandemic attributes, it is important to know the genetics of this thriving population to reduce potential human health risk if newer virulent clones emerge.

To determine the pathogenic potential of environmental *V. parahaemolyticus* isolates, we used Caco-2 cell line assays. As an initial step, the transcriptional activity of the haemolysin and T3SS2 effector genes were analysed in the selected isolates. While all the clinical strains transcribed both the haemolysin genes, only *trh* gene was functional in the environmental isolates. The expression results were substantiated by low haemolytic activity of the environmental isolates on human RBC. It is shown that poor expression of *tdh* gene corresponds to two point mutations in the promoter region (substitution of A to G in the − 35 sequence, G to A at − 3 nucleotide position from the − 10 sequence) [48, 49]. We presume that these mutations may be one of the reasons for the observed reduced transcription. Moreover, we found the isolates carried a single copy of *tdh* (Additional file 1: Figure S4), which is generally responsible for weak and intermediate haemolysis due a weak promoter compared to *tdh2* [48]. The existence of a second copy of *tdh* or mutations in the promoter needs to be further determined. Similarly, the low haemolytic activity of clinical as well as environmental *tdh*^−^*trh*^+^ isolates was not surprising as both contain the *trh2* allele (Additional file 1: Figure S4), which exhibit reduced hemolysis, not due to reduced gene expression, rather, owing to the differences in the amino acid molecular conformation and binding capacity of RBC by the *trh2* product [50, 51]. Further, the *tdh*-*trh*^+^ environmental isolate K23 transcribed the genes *vopC*, *vopA* and *vopL* encoding effectors that promote invasion and modulate host cell behaviour, making it a possible pathogen. A similar functional T3SS2β was seen in *trh*-positive *V. parahaemolyticus* elsewhere in India [52].

Even though T3SS2α was not functional in *tdh*^+^*trh*^−^ environmental isolates, they were included in downstream analysis to know if the isolates could still induce any pathogenic effect. To begin with, we found all the isolates to be cytotoxic to Caco-2 cells without discernible differences in cytotoxicity between clinical and environmental isolates, as shown previously [53, 54]. Therefore, cytotoxicity may not be a reliable indicator of pathogenesis. It was also found that the ability to induce cytotoxicity was not dependent on the virulence genes tested. Interestingly, in the present investigation, the cytotoxicity of *tdh*^−^*trh*^+^ isolate K23 was very low compared to its clinical counterpart with a similar genetic profile. It is well established that T3SSs are responsible for host cell cytotoxic damage, with T3SS1 being the dominant contributor [55]. Perhaps the isolate might have a varied architecture and integrity of T3SS1 gene cluster causing it to assemble a defective translocon and a reduced delivery of T3SS1 effectors responsible for cytotoxicity. It is also possible that the clinical strain might harbour some exclusive genes not present in environmental isolate genome that can induce cytotoxic damage. Meanwhile, the cytotoxicity of the isolates that do not harbour virulence genes was noteworthy. There may be other yet unidentified genes contributing to host cytotoxicty and isolate-specific variations in the virulence of *V. parahaemolyticus*.

The environmental *V. parahaemolyticus* obtained in the study could adhere to and invade intestinal cell line, irrespective of the virulence profile. The ability of the representative *tdh*-positive (H14) and *trh*-positive (K23) isolates to invade was followed by survival and replication within the intracellular environment. Further, infection with H14 and K23 caused disruption of tight junctions and re-organisation of actin cytoskeleton, both of which can compromise the epithelial integrity. Recent research classifies *V. parahaemolyticus* as a facultative intracellular pathogen and T3SS2 effectors, VopC and VopL promote invasion and intracellular survival [56] in addition to inducing dramatic changes in the actin cytoskeleton along with VopV [57–59]. It was found that in spite of a functional *vopC* and a weakly transcribed *vopL*, H14 could still gain access to Caco-2 cell, persist inside and induce changes on tight junctions and actin cytoskeleton. Burdette et al. [60] showed that T3SS1 also causes changes in actin cytoskeleton. The effects seen with the environmental strains may be due to an active T3SS1 albeit the contribution of T3SS2 effectors cannot be ignored, especially with K23, which can emerge as a potential pathogen and elicit a full blown pathogenic response under a favorable environment of host intestine. Moreover, the study detected a non-toxigenic isolate, ME8, from estuarine water exhibiting an invasive phenotype. Perhaps it may be the result of high levels of adhesion rather than active invasion. Recent studies have identified *tdh*^−^*trh*^−^*V. parahaemolyticus* causing gastroenteritis [61–63], indicating the highly complex nature of the virulence of this pathogen.

## Conclusion

This study is the first to extensively characterise the virulence, genetic diversity and elucidate the in-vitro pathogenic effects of *V. parahaemolyticus* isolated from Indian sub-continent. The results indicate the presence of new *V. parahaemolyticus* having pandemic traits from seafood that can adhere to, invade, replicate and cause structural changes in intestinal cell lines. In vivo studies are required to confirm enterotoxicity of these strains; yet, the demonstrated effects on intestinal cell line indicate their enhanced virulence potential. Attributing pathogenic potential for environmental isolates is an important aspect of understanding health risks associated with seafood borne *V. parahaemolyticus*. Although there have been significant progress in the virulence of *V. parahaemolyticus*, our data clearly indicate the difficulty in discriminating pathogenic and non-pathogenic *V. parahaemolyticus* with the current set of virulence genes, particularly when various pathotypes exist. We believe the concept of virulence in *V. parahaemolyticus* needs to be revisited and investigated for each pathotype. The threat of evolving pandemic and potentially pathogenic *V. parahaemolyticus* “environmental” strains looms on seafood consumers and thus, the need for dedicated *V. parahaemolyticus* surveillance programs in Indian marine and estuarine environment. Moreover, this is a reminder to strongly consider the bacterium in the event of a gastroenteritis outbreak.

## Methods

### Sample collection, processing and identification of *V. parahaemolyticus*

Environmental samples consisting of marine and estuarine water, plankton and different seafood were collected from several sampling sites during the period from 2012 to 2013, covering the coastal areas of Kerala. Samples were processed according to the American Public Health Association protocols [64] (see Additional file 1). For each sample, growth in alkaline peptone water (APW) was sub-cultured on thio-sulfate citrate bile salt sucrose (TCBS) agar (HiMedia, Mumbai, India), incubated for 18-24 h at 37 °C. Presumptive isolates (non-sucrose fermenting green colonies) were identified by PCRs for species- specific *toxR* [65] and *tlh* [66] genes.

### Detection of virulence-associated genes

The identified isolates were tested for the haemolysin genes *tdh* and *trh* [66]. The pandemic strain marker genes were verified by GS- and ORF8-PCR [14, 15]. All the *tdh*- and/or *trh*-positive isolates were further tested for the T3SS genes specific to T3SS1, T3SS2α and T3SS2β (Additional file 1: Table S1). *V. parahaemolyticus* RIMD2210633 served as positive control for *toxR*, *tlh*, *tdh*, T3SS and T3SS2α while ATCC17802 was used as the control for *trh* and T3SS2β.

### Serogrouping

Serological analysis of lipopolysaccharide (O) and capsular (K) antigens of *V. parahaemolyticus* was done using commercially available *V. parahaemolyticus* antisera kit (Denka Seiken, Tokyo, Japan) according to manufacturer’s instructions.

### Pulsed-filed gel electrophoresis (PFGE)

PFGE of 15 *V. parahaemolyticus* toxigenic isolates belonging to diverse serovars was performed according to the standardised PulseNet protocol [67]. *Salmonella enterica* serovar Braenderup strain H9812 was used as a molecular weight marker. The PFGE profile of a clinical O3:K6 pandemic strain IDH2826 was included in the clonal comparison. The PFGE image was captured using a Gel Doc XR system (Bio-Rad Laboratories, Hercules, CA), and the gel image was normalized by aligning the peaks of the *Xba*I (Takara, Shiga, Japan) size standards of S. Braenderup in each gel and was analyzed by using BioNumerics software version 4.0 (Applied Maths, Sint-Martens-Latem, Belgium). The similarities between isolates were evaluated by cluster analysis with the UPGMA method and the Dice correlation coefficient with a position tolerance of 1.5%.

### Reverse transcriptase (RT) PCR

Bacteria were grown in LB broth with 3% NaCl to their mid-logarithmic phase and the total RNA extracted with RNeasy mini kit (Qiagen, Valencia, CA, USA). The extracted RNA was reverse transcribed using High-Capacity cDNA Reverse Transcription Kit (Applied Biosystems, Foster City, CA, USA). The cDNA generated was used as a template for the PCR using respective T3SS primer pairs.

### Caco-2 cell culture

Caco-2 (ATCC HTB37, RIKEN BioResource Center, Tsukuba, Japan) cells were grown in Dulbecco’s Modified Eagle Medium (DMEM, Gibco, Carlsbad, CA, USA) comprising 10% (v/v) Fetal Bovine Serum (FBS, PAN-Biotech, Aidenbach, Germany) and 100 μg/ml penicillin-streptomycin (Gibco). The cells were grown at 37 °C with 5% CO_2_ for 5–7 days.

### Haemolysis assay

Haemolysis assay of the *V. parahaemolyticus* isolates was performed as described previously [53] with slight modifications. Briefly, freshly drawn human erythrocytes were rinsed three times with sterile phosphate buffered saline (PBS) and resuspended in PBS to 4% (v/v). About 100 μl of the suspension was mixed with an overnight culture of bacteria in LB broth-3% NaCl (3 X 10^8^ cfu/ml) and incubated at 37 °C for 6 h and 12 h. Subsequently, the plates were centrifuged and the haemoglobin released was estimated by measuring the absorbance of the supernatant at 415 nm (OD_415_). PBS and 20% (v/v) Triton-X 100 were used to determine the maximum and spontaneous haemoglobin release. The experiment was performed twice with six technical replicates. Percentage of haemolysis was expressed by the formula given below.

$$ \% hemolysis\kern0.5em =\frac{O{D}_{415} for\ experimental\ release-O{D}_{415} for\ spontaneous\ release}{O{D}_{415} for\ maximum\ release-O{D}_{415} for\ spontaneous\ release}\kern0.5em \times 100 $$

### Cytotoxicity test

Bacteria were grown in LB-3% NaCl at 37 °C overnight with shaking. Caco-2 cells were seeded in 96-well plates and grown until confluent. Three different wells with Caco-2 cell monolayers were co-cultured with PBS-washed bacteria at a multiplicity of infection (MOI) of 100:1 for 4 h. The release of lactate dehydrogenase (LDH) into the medium was quantified using Cytotox96 non-radioactive cytotoxicity kit (Promega, Madison, WI, USA), following manufacturer’s instructions. The LDH release (% cytotoxicity) was calculated according to the formula:

$$ \% Cytotoxicity\kern0.5em =\frac{O{D}_{490} for\ experimental\ release-O{D}_{490} for\ spontaneous\ release}{O{D}_{490} for\ maximum\ release-O{D}_{490} for\ spontaneous\ release}\kern0.5em \times 100 $$

The spontaneous release was the amount of LDH released from the cytoplasm of uninfected cells, whereas the maximum release was the amount of LDH released by total lysis of uninfected cells. The assay was replicated three times. A time course analysis of H14 and K23 cytotoxicity was also made at 1, 2, 3, 4, 5 and 6 h post infection.

### Adhesion and invasion assays

Adherence and invasion of *V. parahaemolyticus* were determined simultaneously [68] with modifications. In brief, 5 × 10^5^ Caco-2 cells were seeded and grown to confluence in 24-well tissue culture plates. PBS washed bacteria was added at an MOI of 10:1 to the confluent monolayer and incubated at 37 °C for 2 h at 5% CO_2_. Infected monolayers were washed thrice with PBS and lysed using 0.1% Triton X-100 (Sigma-Aldrich, St Louis, MO, USA). Aliquots of the appropriate dilutions of the lysate were plated on LB with or without 3% NaCl and incubated overnight at 37 °C to count total adherent bacteria (CFU ml^− 1^). Adhesion efficiency was calculated as the total number of bacteria adhered expressed as a percentage of the number of bacteria in the original inoculum. For determining bacterial invasion, the infected monolayers were washed with PBS and further incubated for 1 h in DMEM containing 50 μg/ml gentamicin (Sigma) to kill the extracellular bacteria. After washing thrice, the monolayers were lysed and bacterial cells quantified. For each isolate, invasiveness was measured as the invasion index, which is the number of invaded organisms taken as a percentage of the number of adhered bacterial cells. The experiments were repeated three times and SEM was calculated. *S. enterica* serovar Typhimurium and *E. coli* JM109 were used as positive and negative controls. Isolates were scored as adherent or invasive if their index was more than that of *E. coli* JM109.

For the measurement of bacterial intracellular proliferation, invasion assay was carried out as above. After killing extracellular bacteria, the DMEM + 50 μg/ml gentamicin was removed and DMEM + 10 μg/ml gentamicin was added. At time points following the addition of DMEM-gentamicin mixture, cells were lysed and bacteria quantified. Proliferation was determined by calculating the percentage of the initial colony forming units (the point at which DMEM - gentamicin mixture was added) present at each time point.

### Fluorescence microscopy

Caco-2 cells were seeded onto type I collagen-coated (0.01%, Sigma) coverslips in 6-well plate and grown till confluent. Cells were infected with an overnight culture of bacteria grown in LB broth with 3% NaCl at an MOI of 10:1 and incubated at 37 °C for 2 h. Following infection, coverslips were washed with PBS and fixed in 4% para-formaldehyde, after which the cells were rinsed with PBS supplemented with 0.1% Triton X-100 before being blocked for 1 h with 10% FBS and 0.1% Triton X-100. The cells were incubated overnight with rabbit anti zonula occludin protein 1 (ZO-1, N-term) (Invitrogen, Carlsbad, CA, USA) in blocking buffer. The cells were washed with PBS and labelled with Alexa-Fluor 568 goat anti-rabbit antibody (Molecular Probes, ThermoFisher Scientific, Waltham, MA, USA) in blocking buffer for 1 h. The coverslips were then stained with 4′,6-diamidino-2-phenylindole (DAPI) for 10 min, dried and coated with Fluoromount-G (EMS, Hatfield, PA, USA). For visualizing F-actin, cells were stained with Alexa-Fluor 488 phalloidin (Molecular Probes) after permeabilisation, and counterstained with DAPI. Images were captured with Leica TCS WLL SP8 confocal laser scanning microscope at 40X or 60X magnification with voltage and intensity kept constant and are presented as maximum intensity projections from Z-stacks. Images were processed using ImageJ (NIH).

### Statistics

The significance of differences between groups was assessed using Student’s t-test or one way analysis of variance (ANOVA) with Tukey’s multiple comparison test using GraphPad Prism version 5.0 (GraphPad Software, San-Diego, CA, USA). *p* < 0.05 was considered statistically significant.

## Supplementary information

**Additional file 1: **Supplementary method. **Table S1**. Primers and annealing temperatures (Ta) used to characterize *V. parahaemolyticus* isolates. **Figure S1**. Transcription of *trh*. **Figure S2**. Hemolytic activity of *V. parahaemolyticus* isolates on human RBC. **Figure S3**. Transcription of T3SS genes. **Figure S4**. Partial amino acid sequence alignment of TDH (A) and TRH (B).

## Data Availability

The datasets used and/or analysed during the current study are available from the corresponding author on reasonable request.

## Abbreviations

ANOVA: Analysis of variance
CFU: Colony forming units
GS-PCR: Group specific polymerase chain reaction
LDH: Lactate dehydrogenase
NICED: National Institute of Cholera and Enteric Diseases
OD: Optical density
ORF: Open reading frame
PFGE: Pulsed-field gel electrophoresis
SE: Standard error
T3SS: Type III secretion system
TDH: Thermostable-direct hemolysin
TRH: TDH-related hemolysin
UPGMA: Unweighted pair group method with arithmetic mean
ZO-1: Zona occludens 1
