# Supplementary material for: Genetic and virulence characterisation of Vibrio parahaemolyticus isolated from Indian coast
Source: BMC Microbiol. 2020 Mar 23;20:62. doi: 10.1186/s12866-020-01746-2 (PMC7092547; doi:10.1186/s12866-020-01746-2)
Supplement: Supplementary file 1 — Additional file 1: Supplementary method. Table S1. Primers and annealing temperatures (Ta) used to characterize V. parahaemolyticus isolates. Figure S1. Transcription of trh. Figure S2. Hemolytic activity of V. parahaemolyticus isolates on human RBC. Figure S3. Transcription of T3SS genes. Figure S4. Partial amino acid sequence alignment of TDH (A) and TRH (B). [file 12866_2020_1746_MOESM1_ESM.docx]

**Additional File**

**A comprehensive investigation of virulence and pathogenicity of *Vibrio parahaemolyticus* from Indian coast**

Divya M. P.^1^, Thandavarayan Ramamurthy^2^ and Sabu Thomas^1^*

^1^Cholera and Biofilm Research Lab, Rajiv Gandhi Centre for Biotechnology, Thiruvananthapuram, Kerala, India

^2^ Translational Health Science and Technology Institute, Faridabad, India

*Corresponding author: Sabu Thomas

^1^Cholera and Biofilm Research Lab, Rajiv Gandhi Centre for Biotechnology, Thiruvananthapuram 695 014, Kerala, India

E mail: sabu@rgcb.res.in

Fax: +91 471 2348096

Phone: +91 471 2529521

**Running Title**: Pathogenic *V. parahaemolyticus* from the environment

**Methods**

*Sample processing*

Surface water samples (1L) collected in sterile Nalgene bottles were concentrated by filtration through 0.22µm membrane (Merck Millipore, MA, USA). The bacteria in the membrane was suspended in 2ml sterile 10mM PBS (pH 7.4) and aliquots transferred to alkaline peptone water (APW, HiMedia, India) and incubated for 6-8hrs at 37⁰C and 180rpm. Plankton were harvested by towing a 100µm mesh-size plankton net for 5-7 min at subsurface depth (ca. 0.5m) and a portion of each plankton sample (5 ml) was homogenized and inoculated in APW. Seafood samples (clam, mussel, shrimp, squid, crab, sardine and others) were collected in clean sterile containers either fresh from the landing sites or from retail markets. Surface tissues, gills, and gut of fish and the meat and liquor of shellfish were pooled, blended at high speed and a dilution of homogenate was inoculated in APW. For shrimp, the entire enrichment was used as before. For each sample, one to two loopfuls of APW was sub-cultured to thio-sulfate citrate bile salt sucrose (TCBS) agar (HiMedia, India) and incubated for 18-24h at 37⁰C. Typical green colonies producing cytochrome oxidase were selected for identification.

**Table S1.** Primers and annealing temperatures (Ta) used to characterize *V. parahaemolyticus* isolates

| **Sl No** | **Primers** | **Nucleotide Sequence (5’-3’)** | **Amplicon**  **(bp)** | **T_a_ (°C)** | **Reference** |
| --- | --- | --- | --- | --- | --- |
| 1 | *toxR* | F: GTCTTCTGACGCAATCGTTG  R: ATACGAGTGGTTGCTGTCATG | 368 | 63 | [1] |
| 2 | *tlh* | F: AAAGCGGATTATGCAGAAGCACTG  R: GCTACTTTCTAGCATTTTCTCTGC | 450 | 58 | [2] |
| 3 | *tdh* | F: GTAAAGGTCTCTGACTTTTGGAC  R: TGGAATAGAACCTTCATCTTCACC | 269 | 58 | [2] |
| 4 | *trh* | F: TTGGCTTCGATATTTTCAGTATCT  R: CATAACAAACATATGCCCATTTCCG | 500 | 58 | [2] |
| 5 | GS (*toxRS*/new) | F: TAATGAGGTAGAAACA  R: ACGTAACGGGCCTACA | 651 | 45 | [3] |
| 6 | *orf8* | F: GCATACAGTTGAGGGGAAAG  R: AGCGCTCTTTGTTTTCTATATG | 1058 | 53 | [4] |
| T3SS1 | | | | | |
| 7 | VP1670  (*vscP*) | F: ACCGATTACTCAAGGCGATG  R: TACGTTGTTGGCGTGATTGT | 392 | 60 | [5] |
| 8 | VP1686  (*vopS*) | F: CAAAAGCGATCACAAAAGCA  R: AGCGACTTAACGGCATCATC | 283 | 60 | [5] |
| 9 | VP1689  (*vscK*) | F:AAGGTTGGCAAAAAGCGTTA  R: GCTGTTCAACGAGCCAAGAG | 192 | 60 | [5] |
| 10 | VP1694  (*vscF*) | F: ACGATGCGACCAACAGTGTA  R: TTTTAATTGCATCGGTGACG | 96 | 60 | [5] |
| 11 | VPA0450 | F: AAGGCTCTGATGTGCTG  R: TGGTTTGGTGGTTCTGT | 219 | 50 | This study |
| T3SS2α | | | | | |
| 12 | VPA1362  (*vopB2*) | F: CTGCAGGTATCGCATCTTCA  R: TTAGAACCAACCGACGAAGC | 343 | 60 | [5] |
| 13 | VPA1339  (*vscC2*) | F: GATTCGCGGAACTCAAGAAG  R: CTTGTCCGAGATCAACGTCA | 250 | 60 | [5] |
| 14 | VPA1327  (*vopT*) | F: CCTCACGAGACCCAGAAAT  R: TCAAGTAAGGCAGGCACAT | 97 | 60 | [5] |
| 15 | VPA1321  (*vopC*) | F: TGGTTTGGTGGTTCTGT  R: AACTGTCCAAGAGGCGTA | 331 | 50 | This study |
| 16 | VPA1336  (*vopZ*) | F: TTTATTCCGTCATCCTAC  R: CTAATCTAACTTTCACCC | 158 | 45 | This study |
| 17 | VPA1346  (*vopA*/ *P*) | F: AGAGGATACAACCGCCAGAT  R: AACGGAAACGAGCAAACC | 358 | 56 | This study |
| 18 | VPA1370  (*vopL*) | F: ACCATCAGCGTCAACAAG  R: ATCGGTAGTCGTAAATAGGC | 502 | 52 | This study |
| T3SS2β | | | | | |
| 19 | *vscC2* | F: GTACTTTGCTGTCTAACC  R: CTTACTCTTAACCTTCCGACG | 1400 | 55 | [5] |
| 20 | *vopB2* | F: GAGCCTGTTGCTCTATGGAGCCAGG  R: CGACACAGAACGCAATGCTTGCTCG | 942 | 55 | [5] |
| 21 | *vopC* | F: AACCAACTTGCGACTAAATC  R: TCCCGACAGTTTTTCTGCAC | 594 | 55 | [5] |
| 22 | *vscS2* | F: TTGATGTTGTTTCGGCTAGC  R: CCACCGCCGAACTCGGCTAACAAG | 224 | 55 | [5] |
| 23 | *vopA* | F: GGTTGTGGAGGTATTCA  R: ATCAAGGAGGGAGGTAA | 359 | 48 | This study |
| 24 | *vopL* | F: CAACTAATGGAGGAGAT  R: CTGTCAGAACGGAATAT | 686 | 50 | This study |

**References**

1. Kim YBU, Okuda JUN, Matsumoto C, Takahashi N, Hashimoto S, Nishibuchi M. Identification of *Vibrio parahaemolyticus* strains at the species level by PCR targeted to the *toxR* gene. 1999;37:1173–7.

2. Bej AK, Patterson DP, Brasher CW, Vickery MCL, Jones DD, Kaysner CA. Detection of total and hemolysin-producing *Vibrio parahaemolyticus* in shellfish using multiplex PCR amplification of *tl*, *tdh* and *trh*. J Microbiol Methods. 1999;36:215–25.

3. Matsumoto C, Okuda J, Ishibashi M, Iwanaga M, Garg P, Rammamurthy T, et al. Pandemic spread of an O3: K6 clone of *Vibrio parahaemolyticus* and emergence of related strains evidenced by arbitrarily primed PCR and *toxRS* sequence analyses. J Clin Microbiol. 2000;38:578–85.

4. Laohaprertthisan V, Chowdhury A, Kongmuang U, Kalnauwakul S, Ishibashi M, Matsumoto C, et al. Prevalence and serodiversity of the pandemic clone among the clinical strains of *Vibrio parahaemolyticus* isolated in southern Thailand. Epidemiol Infect. 2003;130:395–406.

5. Noriea NF, Johnson CN, Griffitt KJ, Grimes DJ. Distribution of type III secretion systems in *Vibrio parahaemolyticus* from the northern Gulf of Mexico. J Appl Microbiol. 2010;109:953–62.


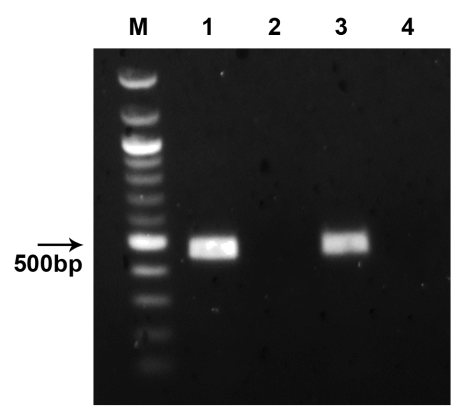


**Figure S1**. Transcription of *trh*. Lane M-100bp ladder, Lane 1-ATCC17802, Lane 3-K23, Lanes 2 and 4-RT^-^ controls


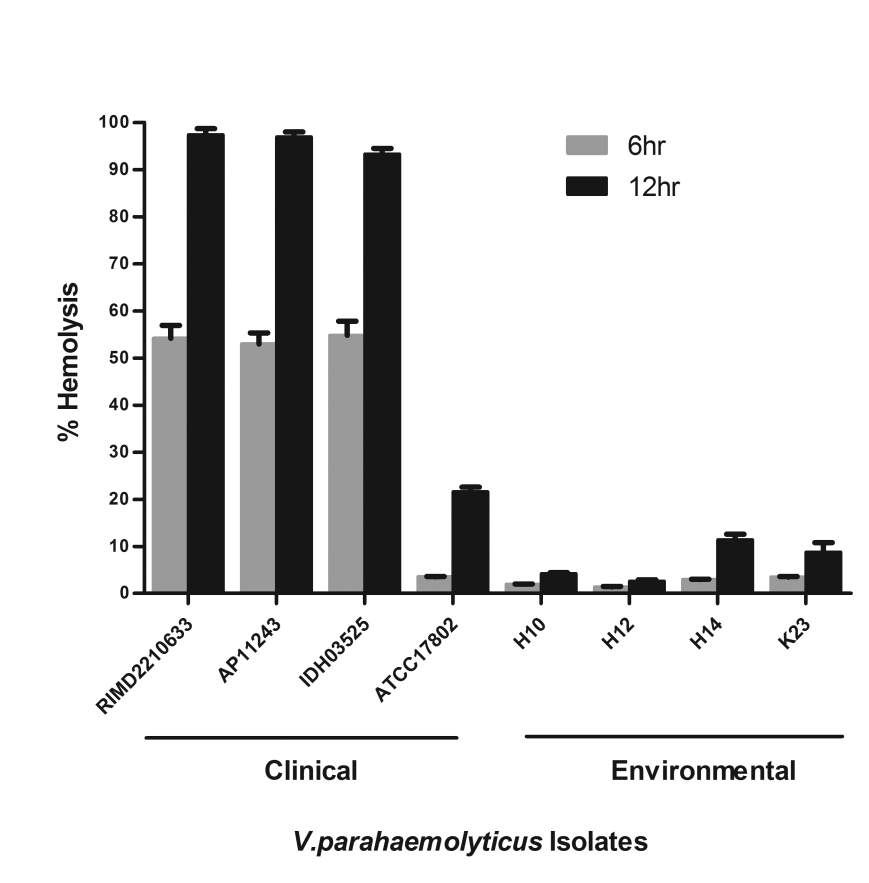


**Figure S2**. Hemolytic activity of *V. parahaemolyticus* isolates on human RBC. The results represent mean ± SE of two independent experiments, with each performed six times.


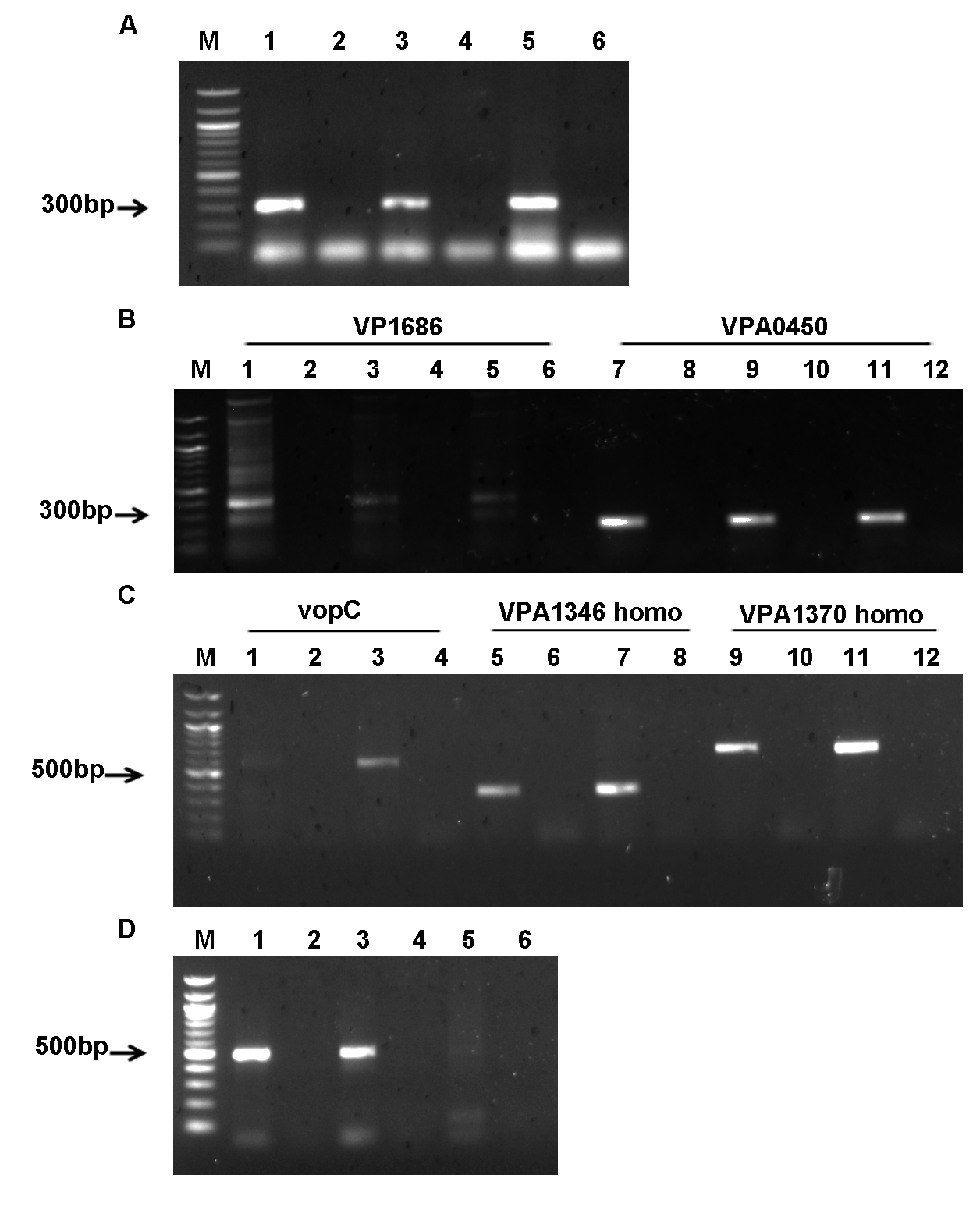


**Figure S3**. Transcription of T3SS genes. **(A)** *gyrB* housekeeping gene. Lanes 1, 3, 5-RIMD2210633, H14, K23, Lanes 2, 4, 6-RT^-^ control. **(B)** T3SS1 genes. Lanes 1, 7-RIMD2210633, Lanes 2, 4, 6, 8, 10, 12-RT^-^ control, Lanes 3, 9-H14, Lanes 5, 11-K23. **(C)** T3SS2β genes. Lanes 1, 5, 9-ATCC17802, Lanes 2, 4, 6, 8, 10, 12-RT^-^ control, Lanes 3, 7, 11-K23. **(D)** T3SS2α VPA1370 gene. Lanes 1, 3, 5-RIMD2210633, AP11243, H14, Lanes 2, 4, 6-RT^-^ control. Lane M in all the gels corresponds to 100bp ladder.


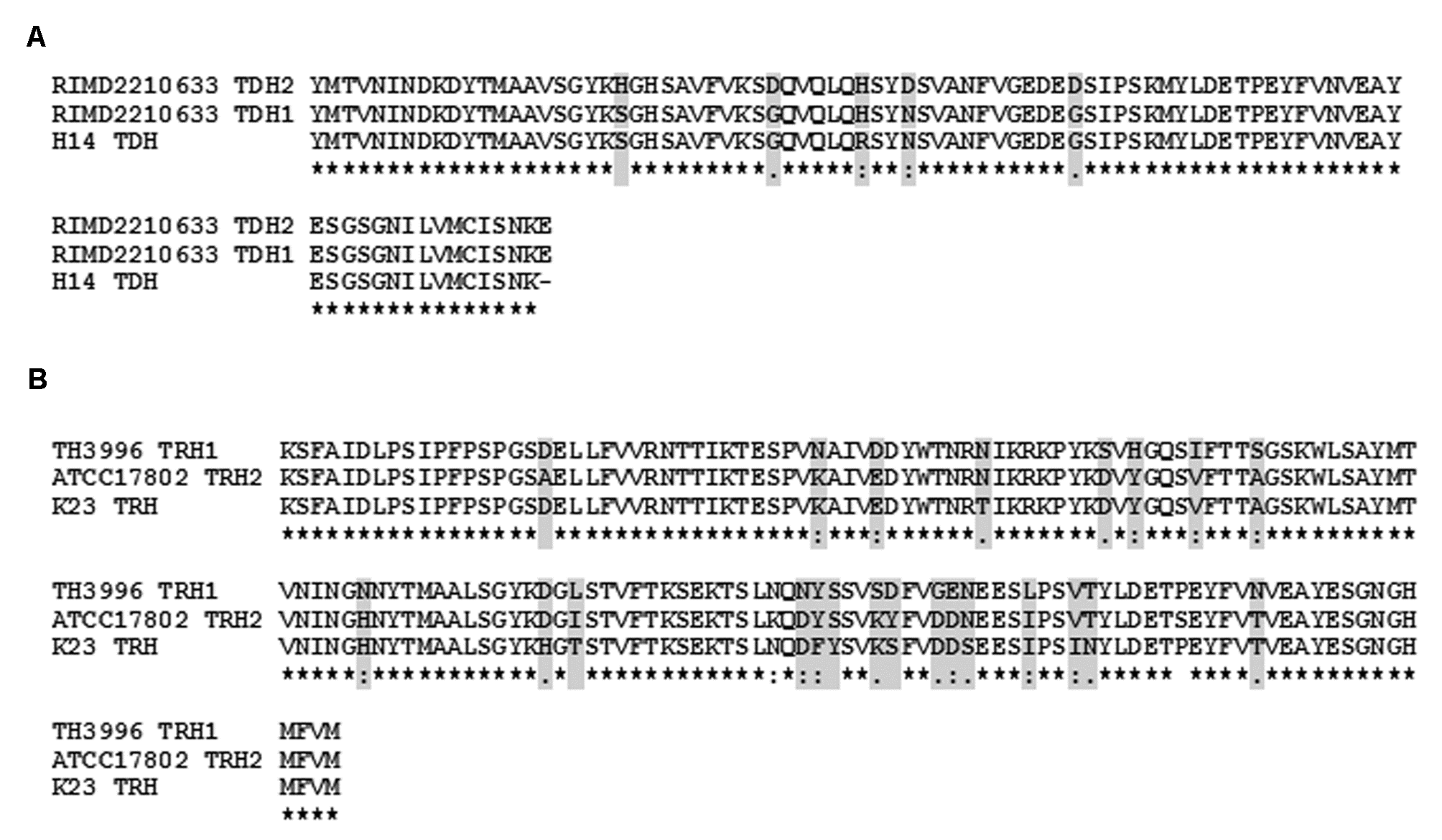


**Figure S4**. **(A)** Partial amino acid sequence of *V. parahaemolyticus* H14 TDH as compared with that of TDH1 and TDH2 of *V. parahaemolyticus* RIMD2210633. **(B)** Partial amino acid sequence alignment of TRH of *V. paraheamolyticus* TH3996, ATCC17802 and the isolate K23. Dots represent identical amino acids.
